# Book Notices

**Published:** 1896-10

**Authors:** 


### Book Notices.

MEDICAL JURISPRUDENCE, FORENSIC MEDICINE AND TOXICOLOGY.—By R. A. Witthaus, A. M., M. D., and Tracy C. Becker, A. B., LL. B., and a staff of collaborators. In four royal octavo volumes. Volume III, Forensic Medicine (continued). New York: William Wood and Company. 1896.

In this volume of this excellent work the general subject of Forensic Medicine, commenced in volume II, is continued. The first article is by Dr. J. H. Woodward. on the subject of the "Medico-Legal Relations of Vision and Audition, and of Injuries to the Eye and Ear." In this article the author discusses normal vision, the methods of testing the vision, its acuteness, variations from the normal, etc.; also simulated blindness, injuries of the orbit, injuries and wounds of the eyelids, injuries and wounds of the eyeball, foreign bodies in the eyes, sympathetic diseases of the eye, and injuries of the ear.

Since any defect of sight or hearing, either real or feigned, may play an important part in many law-suits, this subject is a most interesting one to the physician, and especially to the expert; and the masterly manner in which the subject has been handled by Dr. Woodward in this volume will be duly appreciated by the general medical profession.

The second paper in this volume is on the important subject of "The Medico-Legal Aspect of Insurance," by David Murray, Esq., and G. J. Edwards, Esq., of the New York bar, both of them experts in this line. This article covers only about forty pages of the book, but the medico-legal aspect of both life and accident insurance is carefully considered in all of its bearings.

The next article, on the "Medical Aspects of Insanity in its Relations to Medical Jurisprudence," is by Dr. Edward D. Fisher, of New York. Dr. Fisher's reputation in this branch of medicine is well known to the profession of this country, and in this paper he has sustained the enviable reputation already earned. This is the largest article in the book, occupying over two hundred pages, and treats of insanity in all of its forms as bearing on medical jurisprudence. The author's definition of insanity, his description of its causes, symptoms, etc., his classification and description of its different forms, are all excellent. He gives a report of cases coming under the different forms of insanity, illustrating the peculiar symptoms, delusions or hallucinations of each division, and indicating the particular line in which crime, in each class, is more likely to develop.

The next article, occupying about one hundred and fifty pages, is by Tracy C. Becker, Esq., one of the editors of this work, on the subject of "Mental Unsoundness in its Legal Relations." This paper considers the matters of contracts, deeds, wills, etc., by the mentally unsound, in civil law; and of mental unsoundness as defense to crime, in criminal law. In the latter the responsibility of the mentally unsound criminal, whether he is simply eccentric, an epileptic, partially insane, laboring under excitement or passion, impulsive insanity, melancholia or mania, delirium from fever or medicine, kleptomania, delusional insanity, ixtoxication, delirium tremens, dipsomania, etc., is fully discussed, and many cases cited in evidence.

The last section in the book is by Goodwin Brown, on the "Care and Custody of Incompetent Persons and their Estates." This paper, after giving the usual course pursued in the trial and commitment of the insane person, and the legal custody of his property, gives a digest of the statutes of all the States relating to the care and custody of incompetent persons and their estates, and the commitment and confinement of insane persons. The matter contained in this article, containing as it does these laws of the different States, is a most important and useful one to both the medical and the legal professions.

The volume closes with a table of the cases cited in the volume.

Altogether this volume is not one whit behind the very excellent ones of this series which have preceded it, and the publishers' promises have been fully sustained. S. E. H.

---

Anatomy, Descriptive and Surgical.—By Henry Gray, F. R. S., Lecturer on Anatomy at St. George's Hospital, London. New and thoroughly revised American edition, much enlarged in text, and in engravings both colored and black. In one imperial octavo volume of 1239 pages, with 772 large and elaborate engravings on wood. Price of edition with illustrations in colors: Cloth, $7.00; leather, $8.00. Price of edition will illustrations in black: Cloth, $6.00; leather, $7.00. Lea Brothers & Co., Publishers, Philadelphia and New York, 1896.

In our student days we thought the one medical text-book that had reached perfection was "Gray's Anatomy." It spoke so positively, its descriptions were so clear-cut and so easily understood, its illustrations were marvels of clearness and accurateness. But in this age of progress we find new things even in anatomy, and "Gray" has been revised and improved from time to time. We now have a new and thoroughly revised American edition. This revision has been made exclusively by American anatomists—the first in the history of this great text-book on anatomy. It is adapted to the most modern teaching methods and the requirements of American students, and records the atest advances of anatomical science.

The following statement from the publishers is a concise and accurate description of the more important improvements, hence we reproduce it here:

"There has therefore been effected not only a general revision of the work as a whole, but also entire changes in certain departments in which investigation has been especially active during recent years. The sections which have been rewritten are those on the brain, the teeth, and the abdominal viscera, exclusive of the genito-urinary tract, while those on histology and development—a feature peculiar to 'Gray,' and of obvious value—have been remodeled.

"The splendid series of illustrations which have always distinguished 'Gray,' has been enriched in this new edition by no less than one hundred and thirty-five additional engravings. These illustrations have long been known as the most effective and intelligible presentations of anatomical structures, and in the present issue this supremacy is fully maintained.

"The practical application of anatomical facts in medicine and surgery has always been a prominent feature of the work, and this distinctive characteristic has again received the especial care of the editors.

"In short, this edition is presented to the medical public with the confident expectation that it will be found worthy in every respect to maintain the exalted position which the work has for so many years enjoyed as the most convenient and intelligible exposition of its subject."

As every physician knows, the superior character of the mechanical work put on this book in the past it is hardly necessary to state that in this respect—the paper used, the printing, presswork and binding in this edition has never been excelled.

S. E. H.

---

INFANTILE MORTALITY DURING CHILDBIRTH AND ITS PREVENTION.—By A. Brothers, B. S., M.D., Visiting Gynecologist to Beth Israel Hospital, New York; Attending Gynecologist to the New York Clinic for Diseased Women; Instructor in Operative Gynecology at the New York Post-Graduate School, etc., (being the Wm. Furness Jenks Prize Essay of the College of Physicians of Philadelphia). 8vo., cloth, pp. 179. Price, $1.50. P. Blakiston, Sons & Co., 1012 Walnut St., Philadelphia, Publishers, 1896.

Of the six essays entered in the contest for the Wm. F. Jenks Memorial Prize, this was awarded, by the committee, the prize of five hundred dollars. As the other essays were doubtless meritorious ones, the author of this one is to be congratulated on his success.

To the general practitioner this book, giving, as it does, the advances made in recent years in the management of labors, and the author's ten years experience in active midwifery practice, both in hospital and private work, will be found a most interesting and instructive one. The author points out the causes most likely to result in the death of the infant and gives the surest means at our command for preventing the development of these causes, and for the management of the case to a successful issue in those cases in which they do develop. He emphasizes the accouchuer's responsibility to the child as well as to the mother, maintaining that the obstetrician of to-day, besides being well educated, should be a man of sound judgment, conservative, patient, etc., and "must not shrink, in cases of necessity, from the thoughts of version, forceps, symphyseotomy or Cæsarean section."

The book possesses many points of merit, and a careful study of it will be helpful to many. S. E. H.

---

A Manual of Syphilis and the Venereal Diseases.—By James Nevins Hyde, A. M., M. D., Professor of Skin and Venereal Diseases, Rush Medical College, and Frank H. Montgomery, M. D., Lecturer on Dermatology and Genito-Urinary Diseases, Rush Medical College. Octavo, 618 pages, with 44 illustrations in the text and 8 full-page plates in colors and tints. Price, $2.50 net. Philadelphia: W. B. Saunders, 925 Walnut st. 1895.

The subjects discussed in this volume are always interesting to the medical practitioner. Like the poor, these venereal cases "are always with us."

In this volume the authors have succeeded well in providing a convenient epitome of the subjects under discussion. The subjects discussed embrace the three principal venereal diseases, syphilis, chancroid and gonorrhœa, and in addition to these and their complications, balanitis, balano-posthitis, phimosis, paraphimosis, venereal warts, herpes progenitalis and hypochondriasis receive a full and careful investigation.

The authors have wisely avoided in the main the discussion of mooted questions, confining themselves to what is known, and as a result the book is of vastly more worth to the student than one entering upon lengthy discussions of theories. The numerous illustrations will serve an excellent purpose in the matter of making diagnosis more easy. S. E. H.

MANUAL OF THE PRACTICE OF MEDICINE. By George Roe Lockwood, M. D., Professor of Practice in the Woman's Medical College, and in the New York Infirmary; Attending Physician to the Colored Hospital and to the City (late Charity) Hospital; Pathologist to the French Hospital, etc. 935 pages, with 75 illustrations in the text and 22 full-page colored and half-tone plates. Price, $2.50 net. Published by W. B. Saunders, 925 Walnut st., Philadelphia. 1896.

This book is not to be classed with the ordinary compend or manual. It covers the entire range of medical practice, not comprehensively, but in a concise manner, giving most of that which is of value, and avoiding long discussions and much disputed questions. The book has been almost universally commended by the medical press, its thoroughly practical character and the author's success in compressing the greatest amount of practical and useful information into the smallest possible space receiving especial mention and praise. It is fully up to date in every respect, giving the latest accepted views on the etiology, pathology and treatment of disease.

The publisher has done his work well, the book being finely printed and beautifully illustrated throughout. It is surprising how so valuable and so large a book can be published in a style so superior for the very low price of $2.50. S. E. H.

